# Performance Comparison of Multipixel Biaxial Scanning Direct Time-of-Flight Light Detection and Ranging Systems With and Without Imaging Optics

**DOI:** 10.3390/s25103229

**Published:** 2025-05-21

**Authors:** Konstantin Albert, Manuel Ligges, Andre Henschke, Jennifer Ruskowski, Menaka De Zoysa, Susumu Noda, Anton Grabmaier

**Affiliations:** 1 Fraunhofer Institute for Microelectronic Circuits and Systems, 47057 Duisburg, Germany; 2 Department of Electronic Science and Engineering, Kyoto University, Kyoto 615-8510, Japan

**Keywords:** LiDAR, lens-less, direct time-of-flight, full solid state, biaxial scanning

## Abstract

The laser pulse detection probability of a scanning direct time-of-flight light detection and ranging (LiDAR) measurement is evaluated based on the optical signal distribution on a multipixel single photon avalanche diode (SPAD) array. These detectors intrinsically suffer from dead-times after the successful detection of a single photon and, thus, allow only for limited counting statistics when multiple returning laser photons are imaged on a single pixel. By blurring the imaged laser spot, the transition from single-pixel statistics with high signal intensity to multipixel statistics with less signal intensity is examined. Specifically, a comparison is made between the boundary cases in which (i) the returning LiDAR signal is focused through optics onto a single pixel and (ii) the detection is performed without lenses using all available pixels on the sensor matrix. The omission of imaging optics reduces the overall system size and minimizes optical transfer losses, which is crucial given the limited laser emission power due to safety standards. The investigation relies on a photon rate model for interfering (background) and signal light, applied to a simulated first-photon sensor architecture. For single-shot scenarios that reflect the optimal use of the time budget in scanning LiDAR systems, the lens-less and blurred approaches can achieve comparable or even superior results to the focusing system. This highlights the potential of fully solid-state scanning LiDAR systems utilizing optical phase arrays or multidirectional laser chips.

## 1. Introduction

Pulsed light detection and ranging (LiDAR) is a 3D imaging technology based on measuring the time-of-flight (ToF) of a laser signal that is emitted, reflected by the scene, and subsequently detected. Single photon avalanche diodes (SPADs) are particularly well suited for LiDAR applications due to their ability to achieve high timing resolution, sensitivity to low light levels, and the feasibility of implementing array configurations. There are various possible system implementations regarding the emission and reception geometry [1] (pp. 6–8). A camera-like arrangement of the transmission and receiving channel is known as Flash-LiDAR [2,3,4], where the entire scene is illuminated with a spatially broadened laser pulse (flash) and a spatially resolved multipixel detector determines the times-of-flight using imaging optics. The lateral resolution is, therefore, dependent on the number of pixels on the detector chip, similar to a camera. Alternatively, the time-of-flight measurements can also be performed with a spatially narrow laser spot, where only a single point of the scene is illuminated at a time. A 3D image is then created by sequentially scanning the scene. An advantage of this scanning approach is the increased permissible laser power per image point, which has to be distributed across the entire scene in the Flash-LiDAR approach [5]. On the other hand, Flash-LiDAR obtains information about the entire scene with each emitted pulse, while a scanning single-point illumination system must perform a separate measurement for each image point. For real-time applications, this places high demands on the time efficiency of the acquisition of each measurement point. For a trade-off between the permissible laser power per image point and scene acquisition time, the flash approach has been adapted to partially illuminate only scene regions or slices [6,7].

There are different approaches to how the single point scanning LiDAR setup can be implemented. The transmission path can be combined with the receiving path so that both are then scanned jointly (coaxial geometry) across the scene using a mirror construction [8,9,10,11]. Alternatively, only the transmission path is scanned across the scene, while the receiving path continuously observes the entire scene (biaxial geometry). This scanning LiDAR implementation is especially reasonable if the laser source itself is capable of illuminating discrete spots without an external scanning mechanism [12,13,14,15]. These solid-state scanning solutions differ from optical phase array scanning [16,17,18,19] and do not require coherent LiDAR techniques but are compatible with pulsed direct ToF measurements. To ensure that the detector is not confronted with background (ambient) light from all parts of the scene, there are methods that image the scene onto a multipixel detector chip and then only evaluate the pixels where the laser pulse spot is currently imaged on [20,21]. Such a system is to be investigated using a SPAD array with pixel-individual time-to-digital-converters (TDCs) and first-photon measurement architecture [22,23,24,25] as a detector chip. This first-photon measurement means that each pixel can only generate one timestamp per emitted laser pulse. Therefore, a distance measurement with this type of detector typically involves multiple pulses, with the resulting timestamps being evaluated in a histogram. This histogramming allows the time-of-flight of the pulses to be determined even if the detector is triggered additionally by ambient light. While the imaging of the scene allows for rejecting uninteresting parts of the scene containing only disruptive ambient light, using a single pixel per laser pulse is not time efficient for generating a histogram. Therefore, it is investigated whether a blurred imaging of the scene, where the laser spot is smeared over multiple pixels, or even lens-less detection with a bare multipixel chip, would be advantageous.

## 2. Materials and Methods

The LiDAR setups are analyzed with respect to laser power, background light, target distance, and measurement statistics. By considering a broad range of background light intensities, the influence of various environmental conditions and sensor parameters can be addressed, such as dark count rates, sunlight reflections, and other weather-induced constant photon fluxes onto the detector, as all of these are represented by uncorrelated “background” photons. For this purpose, a model for the received light is first developed, based on which a signal-to-noise ratio (SNR) analysis and a Monte Carlo simulation of measurement scenarios are performed. The SNR and the simulations will offer insights into performance differences, including robustness against background light, the minimum required laser power, and the range of the systems.

### 2.1. Rate Model

**Figure 1:**
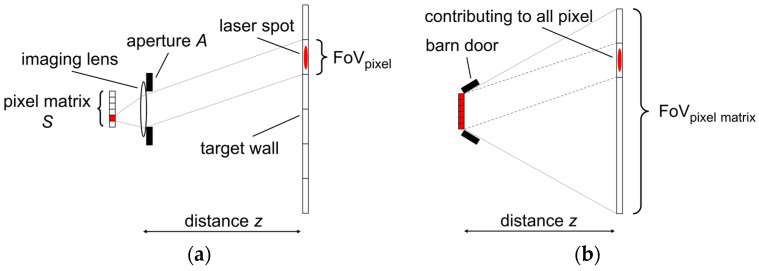
(**a**) Schematic side view of the pixel FoV for the case that the target wall is imaged in focus onto the pixel matrix; (**b**) detector FoV for the lens-less case. The dashed lines indicate the fraction of the wide field of view that is contributing any laser signal. Note that the barndoor aperture depicted is not actually limiting the FoV to the same size as in (**a**), since it is only a scheme.

The model of the received light is designed for the comparison of a focused setup with an aperture area of *A* and a lens-less setup with a multipixel sensor area of *S*. Figure 1 visualizes the contribution of the background light and laser light by the fields-of-view (FoV).

A flat wall at distance *z* is used as the target. The background (ambient) light emitted by the target wall and the reflection of the laser pulse are received by either the aperture and imaging optics sketched in Figure 1a or the bare detector chip as shown in Figure 1b. The detectable intensity is considered to be proportional to the receiving areas *A* and *S*. For simplicity, the rate model is set up only for the central measurement point on the optical axis. Other measurement points would follow a very similar investigation but with an angular-dependent receivable laser intensity due to the Lambertian surface characteristics.

Both systems are designed to have the same total FoV, either by the imaging optic or by barn door covers. While the focused system has equally sized pixel FoVs, the lens-less system covers the same total FoV with every pixel. Unlike in the focused case, where each pixel observes its individual patch of background light and one pixel also observes the laser signal, the lens-less approach results in an identical signal mix for every pixel. The parameters of interest are the photon rates impinging on the detectors. With the knowledge of the laser wavelength, background light spectrum, and detector properties, these rates could be derived from common intensity units. In this paper, the natural unit for the received light will be rates or rates per pixel (Hz). For a detector with *N* pixels, it will be a total background rate per receiving area *R_B_* and a laser signal rate per receiving area *R_L_*. Table 1 shows the resulting impinging rates on the detectors.

The division by *N* of the background rate per pixel originates for the focused setup from the single-pixel FoV, while for the lens-less case, it describes the pixel area fraction of the total receiving area. In contrast to the focused setup, where the laser spot is imaged onto one pixel, the lens-less setup can use all pixels for a measurement. A multiplication by *N* of the lens-less column corresponds to the total received rates. These correlations show that for equal sizes of *A* and *S*, the lens-less approach will receive the same laser signal but gather *N* times more background light. The total rate of the laser signal only depends on the receiving areas.

The transition between these two setups is the blurred imaging of the laser spot onto a pixel region, similar to a “circle of confusion” in traditional imaging optics. The background rate per pixel is independent of the blurring and corresponds to the background rate of the focused setup. The laser signal rate has to be adopted by dividing the focused rate by the number of pixels illuminated by the blurred laser spot. For every degree of blurring, the total received laser rate is still constant for a fixed size of the receiving area. When not indicated differently, the receiving areas *A* and *S* are chosen to be equal throughout this paper. Thereby, the focused and blurred setups are directly comparable to the lens-less setup.

In summary, the background rates per pixel are equal for the three setups. The laser signal per pixel (laser rate) is maximum for the focused setup and must be divided by the number of pixels of the detector for the lens-less setup. For the blurred setup, it must be divided by the number of pixels that are illuminated by the blurred laser spot on the detector.

### 2.2. Probability Density Function

This paper will focus on 2D SPAD array detectors with a first-photon measurement principle, as this sensor architecture is reasonable for biaxial direct time-of-flight-based LiDAR systems [21,22]. The architecture allows for pixel-individual ToF measurements. It also means that for each laser pulse emitted, each pixel can only generate one timestamp. For a reliable distance determination, multiple timestamps might have to be evaluated, since it is a statistical issue whether a background photon generates a timestamp at a random time or a signal photon is measured that contains reasonable timing information. Moreover, as a result of the first-photon measurement principle, the probability for generating a timestamp is not only rate-dependent but also time-dependent as described by the probability density function (PDF) in Equation (1).

(1) $$PDF\left(t\right)=\left\{\begin{array}{ccc} r_{B}\cdot \exp(-r_{B}\cdot t), & & 0\leq t<t_{ToF}\\ (r_{B}+r_{L})\cdot \exp(-r_{B}\cdot t)\cdot \exp\left[-r_{L}\cdot \left(t-t_{ToF}\right)\right], & & t_{ToF}\leq t<t_{ToF}+t_{p}\\ r_{B}\cdot \exp\left(-r_{B}\cdot t\right)\cdot \exp\left(-r_{L}\cdot t_{p}\right), & & t_{ToF}+t_{p}\leq t \end{array}\right.$$

This probability density function results from the fact that for any given time *t*, the probability to detect a photon is given by the instantaneous photon rate *r_B_* or *r_B_* + *r_L_* and the accumulated counter-probability of an earlier SPAD activation, e.g., Equation (2):

(2) $$p\left(t\right)\propto 1-\int_{0}^{t}p\left(\tau \right)d\tau$$

**Figure 2:**
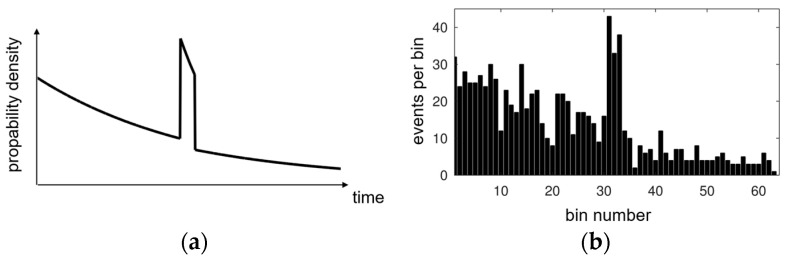
(**a**) Characteristic first-photon probability density function; (**b**) corresponding histogram simulated with 1000 measurements.

The parameters *r_B_* and *r_L_* correspond to the background rate and laser rate impinging the detector as defined in Table 1. The traveling time *t_ToF_* of the laser pulse from transmission via reflection at the target to the reception at the detector is called time-of-flight (ToF). *t_p_* corresponds to the temporal laser pulse width. The temporal laser pulse shape used is rectangular with infinitely steep slopes. The characteristics of this PDF are shown in Figure 2a.

For the simulated ToF measurements, arrival times of incident photons or dark counts are generated from the PDF by the inverse transformation method. Since the arrival times of background events and laser photons follow homogeneous Poisson distributions, their inverted cumulative distribution functions (CDFs) can be calculated separately by following Equations (3) and (4).

(3) $$CDF\left(\tau \right)=\int_{0}^{\tau }PDF\left(t\right)dt=p$$

(4) $$Q\left(p\right)={CDF}^{-1}\left(p\right)$$

(5) $$Q_{background}\left(p\right)=-\frac{ln(p)}{r_{B}}$$

(6) $$Q_{laser}\left(p\right)=-\frac{ln(p)}{r_{L}}+t_{ToF}$$

The final arrival time is the smaller value of both quantile functions in Equations (5) and (6), while the laser photon arrival time is only accepted within *t_ToF_* and *t_ToF_* + *t_p_*. For every laser pulse and pixel, individual arrival times are generated.

### 2.3. Signal-to-Noise Ratio

The constant background rate over time generates an exponentially decreasing signal in the histogram due to the reduced detection probability of a photon to hit a SPAD that was not already triggered before. With an unlimited number of measurements, a histogram would align (proportionally) with the PDF. In real scenarios with limited statistics, a histogram shows more fluctuations, which will eventually prevent the determination of the correct signal pulse position (*t_ToF_*). In the Monte Carlo simulation result shown in Figure 2b based on the displayed PDF, the background and laser rates are chosen to reveal the main characteristics, with 1000 measurements for the sake of clarity.

The evaluation of the setups includes a signal-to-noise ratio analysis similar to the one derived in Ref [26]. The SNR definition used originates from a Poisson-distributed photon statistic and evaluates the number of generated events by the laser signal *N_L_* and the background events *N_B_*:

(7) $$SNR=\frac{\mu }{\sigma }=\frac{N_{L}}{\sqrt{{\sigma_{L}}^{2}+{\sigma_{B}}^{2}}}=\frac{N_{L}}{\sqrt{N_{L}+N_{B}}}$$

The number of background-generated events *N_B_* is evaluated at the same position in the histogram as where the laser pulse would occur. Both numbers of events are obtained by integrating Equation (1) over the temporal laser pulse width *t_p_*. Light hitting the sensor is specified as the background photon rate *r_B_* and the laser photon rate *r_L_*. The SNR includes the number of possible timestamps that can be generated in the histogram, which is the number of laser pulses *M* (accumulated measurements) used for a distance determination in the focused setup:

(8) $${SNR}_{focused}=\sqrt{M\cdot e^{-r_{B}\cdot t_{ToF}}}\cdot \frac{e^{-r_{B}\cdot t_{p}}-e^{-\left(r_{B}+r_{L}\right)\cdot t_{p}}}{\sqrt{1-e^{-\left(r_{B}+r_{L}\right)\cdot t_{p}}}}$$

For the lens-less setup, with every laser pulse, N (number of pixels illuminated) timestamps can be generated, leading to *N* times *M* possible events. Assuming again that *A* has the same size as *S* (compare Table 1), the SNR for the lens-less setup carries an additional reciprocal factor *N* for the laser signal rate:

(9) $${SNR}_{lens\text{-}less}=\sqrt{N\cdot M\cdot e^{-r_{B}\cdot t_{ToF}}}\cdot \frac{e^{-r_{B}\cdot t_{p}}-e^{-\left(r_{B}+\frac{r_{L}}{N}\right)\cdot t_{p}}}{\sqrt{1-e^{-\left(r_{B}+\frac{r_{L}}{N}\right)\cdot t_{p}}}}$$

This SNR definition in Equation (9) will be used throughout this paper since it describes the focused, blurred, and lens-less setup with a specific choice of *N*.

### 2.4. Laser Pulse Detection Probability

**Figure 3:**
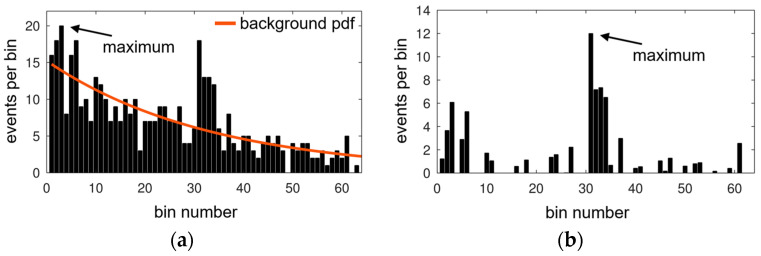
(**a**) Measurement histogram and trigger probability of the background light (red curve); the maximum position at early times does not correspond to the laser signal that is located in bin 31. (**b**) The same histogram with subtracted background characteristic and arrow-indicated laser signal contribution resulting in a maximum at the correct position.

Since a scanning LiDAR system might use only a few laser pulses, the applicability of the SNR is to be verified in this paper. A simulation of measurements and their probability of a successful distance determination is used for further investigation of the performance differences. This probability describes the chance of a specified algorithm to extract the correct ToF (distance) out of the histogram. Since the ToF is the position of the laser pulse characteristic within the histogram, this probability is referred to as laser pulse detection probability (LPDP). The algorithm receives the histogram and subtracts the background characteristic that is assumed to be known. Then, the (first global) maximum of the resulting histogram is defined as the ToF measurement result, as illustrated in Figure 3.

The simulations include a finite time resolution and a temporal laser pulse width so that a measurement is considered successful if the histogram maximum occurs within *t_ToF_* and *t_ToF_* + *t_p_*. The pulse width is 3 ns, which is comparably short for a pulse-on-demand laser diode. For the discrete time binning, an arbitrary but realistic value of 1 ns bin-width (corresponding to a depth resolution of 15 cm) was chosen. For the numerical simulations shown here, these values have been chosen for simplicity and clarity because larger pulse width or smaller time bins would result in a smearing of the laser pulse that counteracts the highest possible maximum formation in the histogram. Depending on the *t_ToF_*, the laser pulse might be positioned in the regime of three or four time bins of the histogram.

## 3. Results

The figures in this chapter evaluate the SNR and the LPDP at a fixed time-of-flight corresponding to an object distance of ten meters when not indicated differently as for the range analysis in Section 3.3.

### 3.1. SNR Analysis

**Figure 4:**
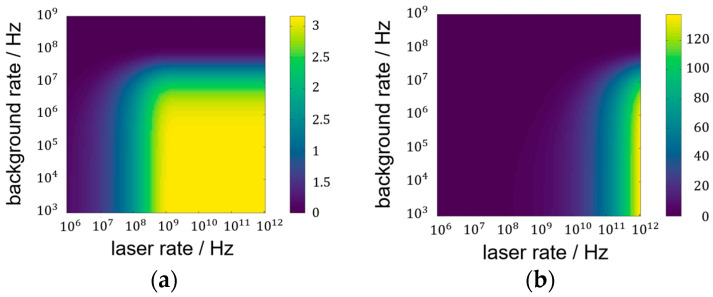
(**a**) SNR result in false-color representation of the focused setup for 10 pulses per measurement at 10 m target distance; (**b**) SNR of the lens-less setup using all 3000 pixels of the detector.

At first, the theoretical SNR is evaluated for an accumulation of ten laser pulses per measurement. In the later analysis, fewer pulses are used to reflect the limited time budget in scanning LiDAR setups. Using ten pulses, however, suggests a higher significance of the SNR since it represents a statistical value. The SNR value is displayed in Figure 4 for six orders of magnitude in laser rate (horizontal axis) reaching the detector. The vertical axis corresponds to the background rate impinging on the detector.

The individual color scale of Figure 4a,b indicates the major difference between the focused setup and the lens-less setup. The focused setup forms a plateau with a maximum SNR of around three for higher laser rates than approximately 10^9^ and smaller background rates than 10^7^. In contrast, the lens-less SNR increases for the depicted laser rates and surpasses the focused SNR maximum by far. Higher SNR values are, in general, favorable for further data processing but do not necessarily lead to better results in LiDAR measurements: The LiDAR measurement result is rated by the chance of extracting the correct time-of-flight from the histogram data. When a measurement setup guarantees this successful time-of-flight extraction, higher SNR values do not produce better results. Therefore, the minimum SNR value that leads to a successful measurement is of interest for a system comparison.

**Figure 5:**
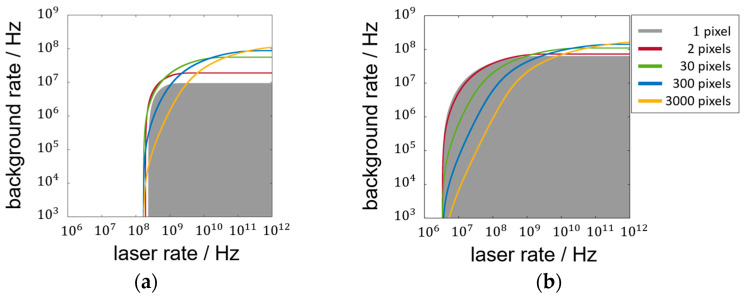
(**a**) Regimes of SNR ≥ 1 for 2 pulses per measurement for focused and blurred laser spots on 2, 30, 300, and 3000 pixels; (**b**) for 100 pulses per measurement.

Since no absolute SNR value can be defined that corresponds to a specific success rate of measurement for varying setups and parameters, the SNR value of 1 is used as a lower limit throughout this discussion. This SNR limit is shown in Figure 5 for the transition from a focused laser spot (1 pixel) to blurring the laser spot (2–300 pixels) to lens-less operation (3000 pixels). The gray area indicates the parameter space of background rates and laser rates where the focused setup surpasses the SNR limit. The colored curves only highlight the outer boundary of the corresponding areas that are not colored completely for visibility.

Depending on the number of pixels the laser spot is blurred on, the parameter space fulfilling the SNR limit changes, and, depending on the impinging background and laser rates, an optimum of the blurring parameter *N* between the boundaries *N* = 1 and *N* = *N_max_* might occur. However, as the ambient parameters usually vary in real environments, there is not just one optimizing blur. With more pixels illuminated, the SNR limit of 1 can be reached at higher background rates with correspondingly high laser rates. For midrange background rates, the minimum laser rate must be increased compared to the focused setup. The extent of these two observations changes to be less advantageous in Figure 5b with more pulses per measurement. There, the maximum background rate reached by the SNR-limited areas increases only slightly with the pixel number. Also, the need for higher laser rates is more pronounced in Figure 5b, visible, for example, for the yellow lens-less curve that separates a larger gray area to its left, as in Figure 5a. This suggests that the potential performance gain of unfocused systems (blurred) is maximized in one-shot scenarios, as will be confirmed by the LPDP results.

The curves in Figure 5 are calculated assuming the lens aperture and detector area as equal and without transmission losses. A larger receiving area of the focused and blurred setup could be represented by a diagonal shift of the curves to smaller laser and background rates with respect to the lens-less curve (3000 pixels). This shifting representation is based on the proportionality of the impinging rates to the receiving area. Transmission losses of the optics would result in a shift towards the opposite direction.

### 3.2. Laser Pulse Detection Probability Analysis

**Figure 6:**
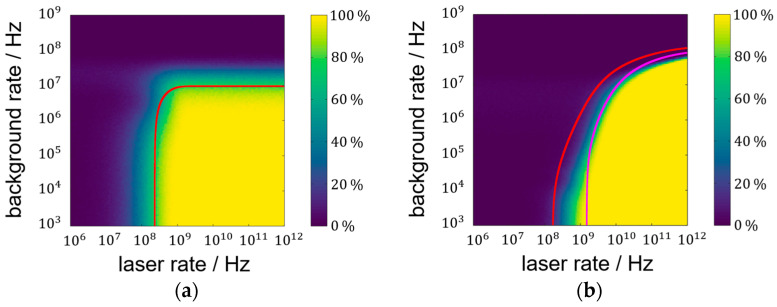
(**a**) Laser pulse detection probability of the focused setup in false-color representation with SNR limit of 1 in red for a two-shot measurement; (**b**) lens-less LPDP result with additional SNR limit curve for SNR = 3 in pink.

Simulated laser pulse detection probabilities (LPDPs) show similar courses like the lower-SNR-limit results. Figure 6 illustrates these similarities by incorporating SNR limit curves into the pulse detection probability maps. While the plateau of high detection probability mimics the SNR limit for the focused setup in Figure 6a, the lens-less setup Figure 6b does not. In Figure 6b, the SNR limit of 3 is drawn to adapt to the lens-less plateau of high probability. Deviations between SNR and simulation are still visible and might originate from the simplicity of the pulse detection algorithm. As defined in the methods section, the algorithm extracts the maximum value of the histogram after background subtraction. The variance in the histogram is proportional to the square root of the histogram-bin value itself. Since the background characteristic is monotonously falling, the maximum variance can be found at early times (beginning of the histogram). The laser pulse characteristic has to, therefore, exceed the early background variance, while the SNR evaluates the signal of the laser pulse and background at the same time position.

**Figure 7:**
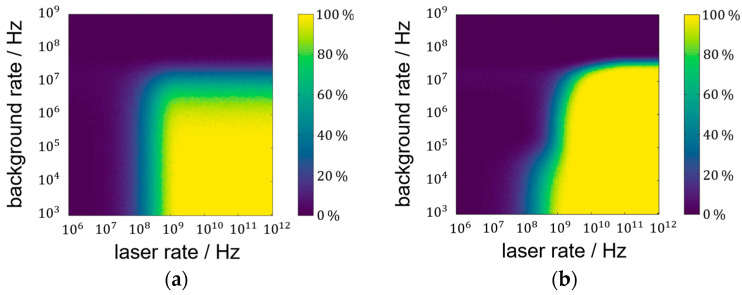
(**a**) LPDP of the focused setup in false-color representation for single-shot measurements; (**b**) LPDP for a blurring onto 30 pixels.

A simulated single-shot measurement is used to compare the focused setup with a blurring onto 30 pixels. While the halved measurement statistic (two shots vs. one shot) significantly affects the measurements, the advantage of a non-focused system at high background rates is clearly visible. LPDP false-color representations for the two setups can be found in Figure 7a,b. The success rate for an LPDP of 90% is limited to a background rate of approximately 24 MHz, which is an order of magnitude higher than for the focused case. Also, the need for higher laser rates at medium background rates is not as pronounced as it is for the completely lens-less case, in agreement with the results for the SNR in Figure 5a.

The use of a single shot instead of two pulses means that the maximum background rate at which a successful measurement is possible is significantly lower for the focused case. A single-shot measurement with a desired pulse detection probability of more than 90% is only possible if the background trigger probability is below 10%. The trigger probability *p_B_* of a background event before the time-of-flight can be calculated from the PDF to the following:

(10) $$p_{b}=1-e^{-r_{B}\cdot t_{ToF}}$$

by integrating Equation (1) from 0 to *t_ToF_*. The corresponding background rate *r_B_* limit for the single-shot scenario is, therefore, approximately 1.6 MHz. For a two-shot scenario, one early background event can still lead to a successful measurement. The background limit is obtained by demanding the squared *p_B_* to equal 10%. The resulting 5.7 MHz more than triples the possible background rate in the focused case, describing the main differences of Figure 6a and Figure 7a. For the sake of completion, the minimum laser rate at negligible background rates must be a factor of two higher for the single-shot scenario than for the two-shot scenario. The calculation is based on the laser trigger counter-probability:

(11) $$\bar{p_{l}}={1-p}_{l}=e^{-r_{l}\cdot t_{p}}$$

solved for $\bar{p_{l}}=0.1$ (one shot) and ${\bar{p_{l}}}^{2}=0.1\;$(two shots). This comparison of single-shot and two-shot scenarios for the focused setup shows these two significant performance losses that are only partially transferable to the blurred setup. While the minimum laser rate at low background rates also doubles for a single-shot measurement, the gain in background saturation rates only rises from 27 MHz to 34 MHz by 26%.

### 3.3. Range Limitations

**Figure 8:**
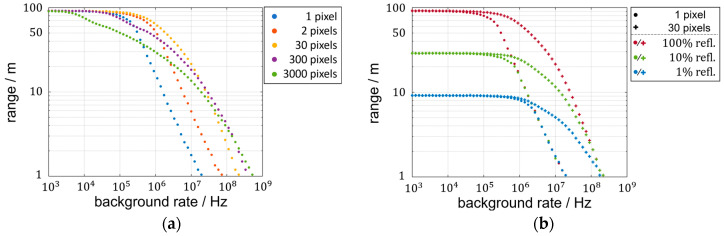
(**a**) Background rate dependency of LiDAR range defined by 90% LPDP for single-shot measurements; (**b**) LiDAR range comparison for focused and blurred laser spots at three target reflectance levels.

**Figure 9:**
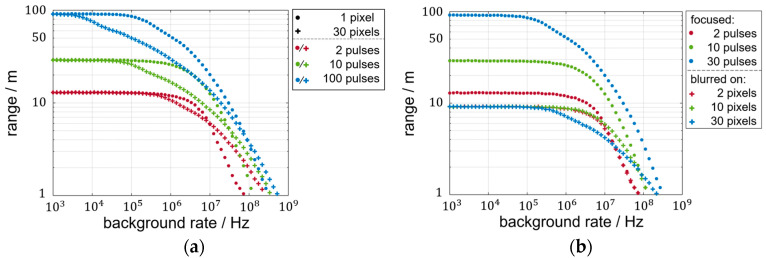
(**a**) Range comparison of the focused measurement and blurring onto 30 pixels for three multi-shot scenarios; (**b**) range results for focused multi-shot scenarios and blurred single-shot scenarios.

The results of the SNR and LPDP so far are based on a fixed-distance evaluation. However, the described characteristics and their distinctiveness depend on the flight time. When discussing the LPDP in the context of achievable ranges, two main aspects contribute to the limitations. On the one hand, the receivable laser rate decreases quadratically with distance. On the other hand, the increase in the time-of-flight increases the chance of background-triggered events before the laser arrival. The latter aspect aggravates further discussion of the LPDP for multiple distances in a false-color representation as seen before. Alternatively, a measurement is defined as successful for LPDP > 90% so that a range value can be assigned to a tuple of laser rate, background rate, and the number of pixels illuminated by the blurred laser spot or 3000 pixels for lens-less operation. For the exemplary range estimation in Figure 8 and Figure 9, the simulated detector properties are chosen to match the characteristics of the CSPAD*alpha* 2D-SPAD detector [22,27] listed in Table 2.

Figure 8a shows the background-dependent course of the range for the maximum laser rate that fulfills the international safety standard [28]. For the 3 ns long laser pulse with a 940 nm wavelength and a pulse repetition rate below 12.5 kHz, the permissible laser peak power is of 31 Watts. For the detector properties shown in Table 2 of a bandpass filter of 10 nm width and an f-number of 1.4, a sunny day (100 klx) would result in approximately 10^8^ Hz of background rate. The length of the histograms used for each data point is adapted to the range value. By this, unusable rear sections of the histograms are avoided that could possibly generate background events that hinder a successful measurement.

The achievable range at sufficiently low background rates is independent of the degree of blurring, as visible in Figure 8a. This is because the decreased probability of a laser trigger event due to the laser rate division is compensated by the gained statistics of more pixels. The probability of no laser events being triggered is, therefore, independent of the number of pixels blurred on. The considered probabilities of zero laser events for a focused and a blurred setup are accordingly equal:

(12) $$\bar{p_{l,N}}={\left({\;e}^{-\frac{r_{l}}{N}\cdot t_{p}}\right)}^{N}={\;e}^{-r_{l}\cdot t_{p}}=\bar{p_{l}}$$

As visible for the data for 300 and 3000 pixels, the limit of negligible background rates decreases for larger numbers of pixels used. This is due to the constant background rate per pixel resulting in a proportional increase in background events with pixel number.

For medium to high background rates, the measurable maximum distance varies with the number of pixels. A conservative blurring onto 30 pixels surpasses the range of the focused setup and a blurring onto 2 pixels for any background rate plotted. When more pixels are used, the range reduction due to the accumulated background events becomes apparent. For high background rates, however, the use of many pixels makes a successful distance measurement possible at all. Taking the achievable range based on the LPDP criterion as a figure of merit, a specific system cannot be optimized by a single degree of blurring.

In Figure 8b, the focused system is compared to the blurring onto 30 pixels for 100%, 10%, and 1% target reflectivity. The system range in the constant regime at low background rates is proportional to the square root of the impinging laser rate, which is proportional to the target reflectivity. The gain in range of the blurred system at higher background rates persists regardless of the reflectivity.

When using more laser pulses per measurement, the general superiority of the blurred setup over the focused setup as achieved in Figure 8b vanishes. The achievable ranges can be compared in Figure 9a again for a blurring onto 30 pixels but for two, ten, and a hundred cumulated laser pulses. The target reflectivity is constant at 1% so that a range of more than 9 m as achieved in Figure 8b originates only from the extra pulses accumulated. With increasing pulse accumulation, the range gain of blurring at high background rates decreases. This can be recognized by the horizontal positions of the intersection where the blurred result surpasses the corresponding focused result. Also, range losses due to blurring, which are not visible in the one-shot scenario in Figure 8a, become already apparent when two pulses instead of one are used. For more accumulation, the range discrepancy begins at lower background rates.

For a clear separation of accumulating pulses in a focused setup and the accumulation of multiple pixels in a blurred setup, Figure 9b depicts three sets of accumulations. The range of the focused setup at low background rates increases with the square root of accumulated pulses. In contrast, the single-shot scenarios are limited in their maximum range and can only reduce the loss in range at high background rates.

## 4. Discussion

The combination of a starring multipixel receiving channel and a scanning transmitting channel bears the weight of signal intensity and ambient light contribution for the detector architecture used. The investigated first-photon measurement architecture of a SPAD array that is limited to a single timestamp per laser pulse and pixel clearly benefits from statistic increasing techniques, such as a blurred imaging optic. Even if the signal distribution onto multiple pixels does not increase the overall range of a LiDAR measurement in ideal conditions, it can maintain the range better under ambient illumination. This extra ambient light robustness decreases with the use of more laser pulses per measurement. In direct ToF LiDAR systems, the single-point scanning systems have the smallest time budget per pixel, making single-shot measurements favorable anyway. The results presented only hold true for first-photon architecture and will differ for multi-event architectures [29,30,31]. Further investigation could elaborate the gain of blurring for these techniques that still have finite event-depths and dead-times.

A lens-less operation that enables the use of all pixels for each measurement independent of the current laser scanning position does not optimize the range. It mostly reduces the range except for situations with extreme ambient illumination. Range reduction in comparison with focused imaging also occurs depending on the degree of blurring. An optimization of the degree of blurring could be performed based on a desired range per ambient illumination profile. Passive adaptation of the degree of blurring to the respective target distance could be achieved by utilizing the distance dependency of the circle of confusion. Active adaptation of the imaging optics could readjust the blurring for varying ambient illumination.

The generalization of the homogenous ambient light distribution within the total FoV in this paper needs to be considered when designing a blurred starring biaxial LiDAR scanner. Parts of the scanned scene exhibiting strong ambient illumination by sunlight or artificial light sources may lead to a 3D image with missing or incorrect spots for a focused LiDAR system. For a blurred system, these spots might be correctly measured due to the enhanced ambient light robustness. In the worst case, these incorrect spots might become even larger since the blurring distributes these bright spots to neighboring pixels.

In the scanning LiDAR implementation discussed, the spatially resolved multipixel detector is not responsible for the spatial resolution of the 3D image since the laser source or laser scanner provides the current angular position. Therefore, the detector can be replaced by a silicon photomultiplier (SiPM) in general. In a blurred scenario, however, the detector area not illuminated by the laser spot cannot be rejected as it can in a SPAD array with pixel-wise TDCs. Future research may investigate biaxial SiPM LiDAR regarding this drawback and possible advantages of threshold-based signal detection compared to the first-photon measurement principle discussed here.

## Figures and Tables

**Table 1:** Comparison of the impinging rates on the detectors.

Light Type	Focused Setup: Rate per Pixel	Lens-Less Setup: Rate per Pixel	Blurred Setup: Rate per Pixel
Background	$r_{B}=R_{B}/N\cdot A$	$r_{B}=R_{B}\cdot S/N$	$r_{B}=R_{B}/N\cdot A$
Laser signal	$r_{L}=R_{L}\cdot A$	${r_{L}=R}_{L}\cdot S/N$	${r_{L}=R}_{L}\cdot A/N_{blurred}$ *

* *N_Blurred_* denotes the number of pixels illuminated by the blurred laser spot.

**Table 2:** Detector properties used for range estimations [22,27].

Parameter	Value	Unit
Receiving area	8.32 × 6.24	mm^2^
Fill factor (with micro lenses)	26.8	%
Quantum efficiency (at 940 nm)	2	%
Avalanche probability	50	%

## Data Availability

The original contributions presented in this study are included in the article. Further inquiries can be directed to the corresponding author.

## Abbreviations

The following abbreviations are used in this manuscript:

LiDAR	Light detection and ranging
SPAD	Single photon avalanche diode
ToF	Time-of-flight
TDC	Time to digital converter
SNR	Signal-to-noise ratio
FoV	Field of view
PDF	Probability density function
LPDP	Lase pulse detection probability
SiPM	Silicon photomultiplier
